# 3D Encapsulation Made Easy: A Coaxial-Flow Circuit for the Fabrication of Hydrogel Microfibers Patches

**DOI:** 10.3390/bioengineering6020030

**Published:** 2019-04-06

**Authors:** Chiara Emma Campiglio, Francesca Ceriani, Lorenza Draghi

**Affiliations:** 1 Politecnico di Milano, Department of Chemistry, Materials and Chemical Engineering “G. Natta”, Via Mancinelli 7, 20131 Milano, Italy; Chiaraemma.campiglio@polimi.it; 2 INSTM—National Interuniversity Consortium of Materials Science and Technology, Via G. Giusti, 9-50121 Firenze, Italy; 3 Politecnico di Milano, Piazza Leonardo da Vinci 32, 20133 Milano, Italy; Francesca.ceriani@mail.polimi.it

**Keywords:** degradable hydrogels, cell delivery, cell encapsulation, microfibers scaffolds, calcium alginate, gelatin, hyaluronic acid

## Abstract

To fully exploit the potential of hydrogel micro-fibers in the design of regenerative medicinal materials, we designed a simple, easy to replicate system for cell embedding in degradable fibrous scaffolds, and validated its effectiveness using alginate-based materials. For scaffold fabrication, cells are suspended in a hydrogel-precursor and injected in a closed-loop circuit, where a pump circulates the ionic cross-linking solution. The flow of the cross-linking solution stretches and solidifies a continuous micro-scaled, cell-loaded hydrogel fiber that whips, bends, and spontaneously assembles in a self-standing, spaghetti-like patch. After investigation and tuning of process- and solution-related parameters, homogeneous microfibers with controlled diameters and consistent scaffolds were obtained from different alginate concentrations and blends with biologically favorable macromolecules (i.e., gelatin or hyaluronic acid). Despite its simplicity, this coaxial-flow encapsulation system allows for the rapid and effortless fabrication of thick, well-defined scaffolds, with viable cells being homogeneously distributed within the fibers. The reduced fiber diameter and the inherent macro-porous structure that is created from the random winding of fibers can sustain mass transport, and support encapsulated cell survival. As different materials and formulations can be processed to easily create homogeneously cell-populated structures, this system appears as a valuable platform, not only for regenerative medicine, but also, more in general, for 3D cell culturing in vitro.

## 1. Introduction

The therapeutic delivery of autologous cells to support the structure and functions of biological tissues is a core paradigm in regenerative medicine. In their infancy, cell-based approaches have been mainly based on the delivery of plain, high-density cell suspensions to the target site. This strategy, however, has generally shown to have a poor clinical outcome, due to major loss of cell viability, and a limited control over cell fate, both in terms of cell differentiation and site-specificity (with cells eventually migrating to other sites) [1,2]. To improve site-specific engraftment of transplanted cells, and achieve to clinically relevant outcomes, in most applications a scaffold or carrier material is essential [3,4,5,6]. 

Among other possible scaffolding options (seeding on preformed 3D porous structures, above all [7,8]), encapsulating cells in short-term degradable hydrogels is a particularly attractive strategy when mechanical properties are not critical. Hydrogels, in fact, offer a highly hydrated environment, and they can adapt to implantation sites and generally maintain graft volume. No less importantly, biological cues can be easily incorporated to be locally released, and to support tissue development [9,10]. Furthermore, as cells are incorporated during scaffold formation, obtaining a uniform distribution throughout the material is generally effortless. 

Nonetheless, when injectable formulations are used for in situ hydrogel formation, an inability to provide functional vascularization at the time of implantation significantly limits the volume of deliverable material. The thickness of the constructs, in fact, is substantially limited to in vivo oxygen, nutrient, and waste diffusion. Although this value significantly depends on the material, microstructures, and the implant site, it generally ranges over a few hundreds of microns, as most hydrogels possess only a nano-scaled porosity [11,12].

To sustain the in vivo survival of encapsulated cells, methods to introduce a higher scale porosity in hydrogels include porogen leaching [13], freeze-drying [14], gas-foaming [15], and rapid prototyping techniques [16]. Due to the presence of potentially detrimental chemicals, or of processing conditions (i.e., temperature and pressure) that are incompatible with cell survival, only a subset of these techniques are practicable when embedded cells are involved. Particulate leaching is among them for selected porogen chemistries, but it has the disadvantage of mainly creating a network of isolated pores. On the contrary, a rapid prototyping technique allows for the creation of complex structures for the organization of tissues and organs [17,18] but, in turn, this requires more complex equipment and fabrication processes.

An interesting possibility for create larger porosities is represented by the fabrication of scaffolds from opportunely assembled hydrogel fibers, where pores are created as spaces between the assembled fibers [19]. Methods that are currently available to prepare hydrogel fibers include 3D-printing [20,21,22], microfluidic devices [23,24,25], and extrusion [26]. While 3D-printing allows for the fabrication of homogeneous fibers over a range of 500 µm, and requires specific equipment, microfluidic channels allow for even smaller fibers (150–500 μm [27]) to be precisely obtained, but with lower yield. The extrusion method, on the other hand, represents the simplest technique for microfiber formation, and involves the injection of a precursor solution into a gelator solution. This fabrication technique is mainly employed to produce continuous single-standing fibers for cell encapsulation [26,28], and mainly relies on the use of sodium alginate and its mild gelation mechanism (room temperature, close to neutral pH) in the presence of divalent cations [29]. Despite its recognized advantages in terms of general biocompatibility and non-immunogenicity, the lack of specific binding sites in alginate for cell adhesion means that it cannot be metabolically dealt with, and the high molecular weight residues are too large for renal clearing. For this reason, chemical modification (e.g., oxidation, or RDG coupling) [30] and blending with cell-interactive components are common strategies [23,31,32,33,34].

Although technologies for hydrogel fiber production have been available for a while, their uses in 3D scaffold have had very limited practical applications, with exceptions being made for 3D-printed structures, that however, are frequently limited in terms of lower fiber dimension.

Here, we proposed a new, extremely simple extrusion system based on coaxial flow, designed to encapsulate cells into self-standing hydrogel patches with controlled fiber diameters. To the best of our knowledge, no previous attempts have been made to control fiber diameters by coaxial-flow in non-microfluidic devices [35], or to fabricate self-standing, cell-loaded scaffolds. 

As the possibility for exploiting this system within different applications relies on the possibility of tuning the chemical composition and properties, blends with common biological macromolecules (gelatin and hyaluronic acid) were also processed to patches, to demonstrate the versatility of this fabrication strategy.

## 2. Materials and Methods 

### 2.1. Materials

All chemicals, if not otherwise stated, were purchased from Sigma-Aldrich, and used without further treatment.

### 2.2. Design of a Coaxial-Flow Fabrication Circuit 

**Figure 1:**
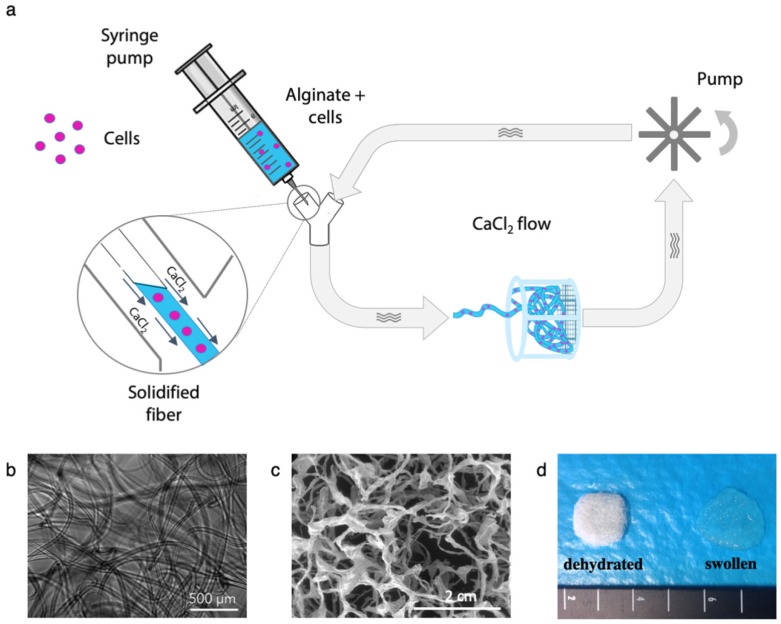
(**a**) Schematic representation of microfiber fabrication system. An alginate-based solution is injected through a syringe pump. The coaxial CaCl_2_ solution flow draws and solidifies a continuous fiber that is collected in the strainer as a cell-loaded hydrogel patch. (**b**) Optical microscope image of the alginate microfibers, (**c**) SEM micrograph of the structures of the freeze-dried fibers, and (**d**) macroscopic appearance of the microfibers patches in their swollen states, and after freeze-drying.

The circuit that was designed for the preparation of continuous alginate microfibers is illustrated in Figure 1a. A centrifugal pump (Sicce Syncra 0.5 to 700 L/h, max head 1.2 mt) circulates a CaCl_2_ solution in a silicon rubber tubing (diameter = 5 mm, thickness = 1 mm) loop. A cell suspension in an alginate solution is injected into the CaCl_2_ flow through a Y-connector using a syringe pump. The coaxial flow draws in the alginate solutions and Ca^2+^ ions crosslinking causes its solidification, resulting in a continuous fiber. The fiber whips, bends, and spontaneously assembles in a fibrous patch, which can be collected from the strainer. 

### 2.3. Plain Alginate Fiber Fabrication and System Optimization 

To prepare the hydrogel fibers, alginic acid sodium salt from brown algae (viscosity 15.0–25.0 cP, Mw = 120–190 kDa) was dissolved in Dulbecco’s Modified Eagle’s Medium (DMEM), and injected in the circulating CaCl_2_ solution. To optimize the fabrication process, and evaluate the effect of solution- and processing-related parameters on fiber formation, the alginate concentration was varied from 1% to 4% (*w*/*v*), and the CaCl_2_ molarity from 35 to 150 mM, and syringe pump advancing rate from 10 to 90 mm/h (1 mm/h, corresponding to 0.3 mL/h). To assess the effects of the syringe needle dimension, 22, 24, and 26 G diameters were compared for the injection of the alginate solution.

For each set of parameters, the extruded fibers were retrieved from the strainer, and their morphologies were evaluated by optical microscopy. Quantitative analyses on fiber diameter were performed on acquired images, using the image-processing software ImageJ [36], based on 30 different measures in random points from at least three different images.

### 2.4. Alginate-Blended Microfiber Production 

To confirm the possibility of creating scaffolds from different compositions, two biological macromolecules were blended with 2% (*w*/*v*) alginate. Gelatin (from porcine skin, Type A) and hyaluronic acid sodium salt (HA, from *Streptococcus* equi, Mw = 1.5–1.8 × 10^6^ g/mol) were blended in at the ratios in Table 1, and processed with the flow circuit, to fabricate microfibers patches.

### 2.5. Rheological Properties

Rheological properties of hydrogel-forming solutions are critical for fiber formation, and hence, they were evaluated using a CVO 120 stress-controlled Rotational Rheometer (Bohlin Instruments, cone/plate geometry with cone diameter = 40 mm and cone angle = 1°). The gap was automatically set to 0.03 mm. A Peltier plate was used to thermostat the solutions at 25 or 37 °C. Steady shear measurements were performed by increasing the shear stress from zero to a maximum of 300 Pa.

### 2.6. Swelling and Weight Loss

Swelling and weight loss of microfibers at 37 °C in phosphate buffer saline (PBS), or in complete cell culture medium were evaluated for up to 21 days, to appraise their kinetics. At selected time-points (1, 2, 3, 5, 7, 24, 48, 72 h, and 7, 14, 21 days), microfibers were strained from the medium, centrifuged to eliminate the excess of water, and weighted with a precision balance. The weight variation (ΔW%) was calculated as:

ΔW% = [(W_t_ − W_0_)/W_0_]100, (1)

where W_t_ is the measured weight of a swollen sample at time t and W_0_ its initial weight.

To increase the stability of the fibers, two different methods were investigated: (i) a post-treatment of fibers in high molarity CaCl_2_ solution (150 mM) for 5 min, and (ii) a combination of external and internal alginate gelation. In this latter case, 5 mM CaCO_3_ was mixed with the cell suspension, and fibers were treated in d-gluconic acid-δ-lactone (GDL, 5 mM, pH 3.4) solution for five seconds.

### 2.7. Cells Encapsulation in Microfibers

For cell experiments, a CaCl_2_ solution was sterile-filtered (0.2 μm), sodium alginate and gelatin powders were disinfected by soaking in ethanol and HA powder, and all of the flow-loop components were sterilized by autoclaving. 

Murine cells L929 (ATCC CCL-1) were detached from flasks with trypsin-EDTA (ethylenediaminetetraacetic acid) when reaching 90% confluence, and they were resuspended (1.5 × 10^6^ cells/mL) in alginate or blended solutions prepared in DMEM. 

After fabrication, cell-loaded patches were placed in cell strainers and cultured in a 12-well multiwell plate with DMEM, supplemented with 10% (*v*/*v*) fetal bovine serum (FBS) with 1% penicillin/streptomycin at 37 °C and 5% CO_2_.

### 2.8. Evaluation of Cell Viability

Cell survival immediately after encapsulation was investigated by double staining with propidium iodide (20 μM) and calcein-AM (2 μM) in a serum-free cell culture medium. After rinsing in PBS, samples were incubated in the staining solution at 37 °C for 40 min and rinsed again before imaging. Fluorescence images were acquired by using a Zeiss Axioplan microscope, using 490/515 nm (excitation/emission) filters for calcein-AM, and 535/617 nm (excitation/emission) for propidium iodide. ImageJ [36] image processing software was used to merge the images and count cells. 

Longer-term viability of embedded cells in alginate-based fibers was measured after 1, 3, 8, and 10 days, using an Alamar Blue^®^ assay according to the manufacturer’s indications. 

### 2.9. In Vitro Cell Release

The number of cells released from the alginate-based patches was estimated by acquiring daily microscopic images of the bottom of the wells, and counting cells in five randomly selected areas (0.7 mm^2^). To evaluate the progression of cell release, strainers containing patches were moved daily to a new plate, in order to account only for cells released in the previous 24 h.

### 2.10. Statistical Analysis

Results are presented as mean ± standard deviation (SD). Statistical analyses were performed using GraphPad Prism software (GraphPad Software Inc., version 6). Statistically significant differences were determined by one-way analysis of variance (ANOVA), followed by Tukey’s post-test for pairwise comparisons, and *p* < 0.05 was considered statistically significant.

## 3. Results and Discussion

### 3.1. Plain Alginate Microfiber Fabrication and System Optimization

The injection of alginate-based solutions in a circulating CaCl_2_ bath was confirmed to be an easy and effective method of preparing alginate-based microfibers, and it proved the effectiveness of the designed flow circuit, as schematized in Figure 1a. For most sets of parameters tested, a continuous fiber was extruded (Figure 1b,c) and, more interestingly, in a subset of cases, it was spontaneously assembled in the strainer as a consistent, self-standing fibrous scaffold, as is shown in Figure 1d, where the appearance of wet and dry microfiber patches is shown. 

**Figure 2:**
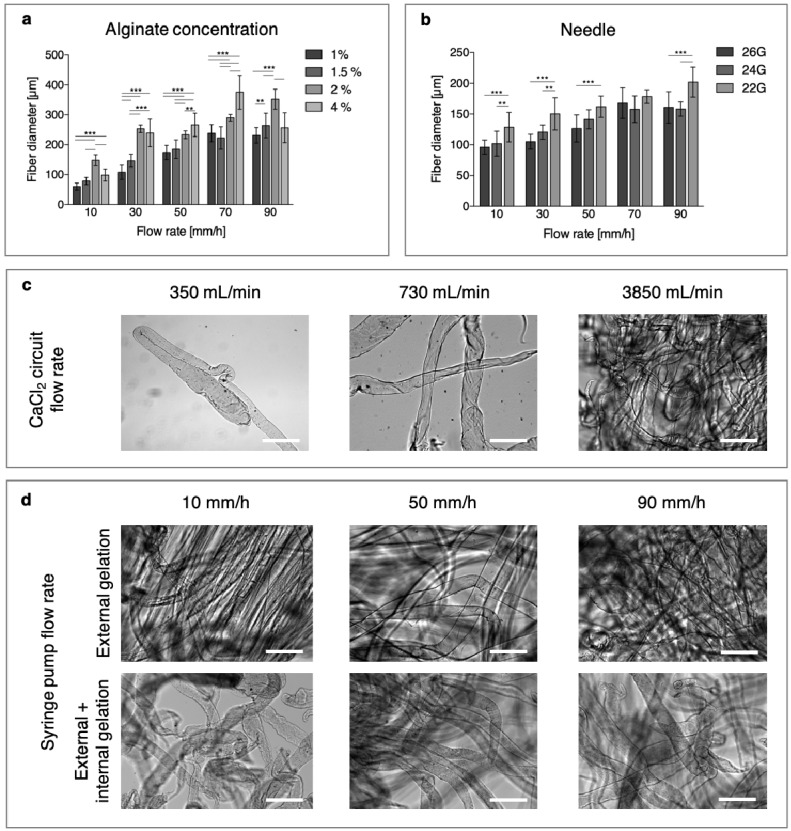
(**a**) Effect of alginate concentration and syringe pump flow rate on fiber dimensions. (**b**) Effect of needle dimension on fiber diameter (* *p* < 0.05, ** *p* < 0.01, *** *p* < 0.001). (**c**–**d**) Qualitative influences of parameters on microfiber dimensions and morphologies: (**c**) effects of CaCl_2_ circuit flow rates and (**d**) effects of alginate solution flow rates. Scale bar = 500 μm.

With the coaxial flow circuit developed, fibers with diameters ranging from 60 to about 400 μm were obtained in a relatively large range of alginate concentrations (Figure 2a). For each concentration, the microfiber diameter was strongly influenced by the syringe pump flow rate, and as reasonable expected, an increase was observed when the solution feed was raised. Increasing the alginate concentration had the same effect for up to 2%, but there was a tendency to a decrease in diameters that was observed for the 4% solutions. This finding is probably due to the high viscosity of the 4% solution, which appeared to have hindered the extrusion of the material, as only short fibers and elongated beads were collected in the strainer. The diameter of the needle also influenced, as expected, the dimensions of the microfibers (Figure 2b). A significant difference in fiber dimensions when using the 22 G or 26 G needle was observed for each pumping flow rate, except for 70 mm/h.

Fibers generally appeared homogeneous in diameter and smooth in their surface, except for some combinations with low CaCl_2_ flow rates (Figure 2c) and low alginate flow rates (10–30 mm/h, Figure 2d), where the fiber surfaces appeared wrinkled.

The flow of CaCl_2_ solution, on the other hand, did not appear to have a distinct influence on the fiber diameter (Supplementary material—Figure S1), but it had a strong effect on the patch coherence, as for the lower-flow speed-separated fibers that were collected. On the contrary, for higher values, a good adhesion among fibers were obtained and coherent scaffolds that were easy to handle, were retrieved. 

When the effect of calcium molarity was investigated, circulating the 150 mM solution resulted in an immediate obstruction of the initial portion of the silicone tubing at high alginate feeding rates, and in needle clogging at lower rates. When using the 70 mM CaCl_2_ solution, fibers were formed, but no adhesion among them was observed in the strainer, possibly due to the saturation of calcium ions in the external alginate shell at the time of collection. As a result, non-consistent and extremely difficult-to-handle structures were retrieved, and 35 mM was chosen for the production of continuous microfibers and coherent, consistent scaffolds.

Results suggest that an effective adhesion between fibers requires crosslinking that should not have proceeded too far as they reach the strainer, because this allows further crosslinking to involve molecules from different fibers, and create strong adhesions among them. For this reason, a low concentration of crosslinking ions and a short permanence in the flow before the collection will result in self-standing, easy-to-handle patches.

The use of a coaxial flow in a non-microfluidic device allows for the production of fiber diameters that are comparable to those obtained in microfluidic devices (150–500 μm [27]), but with a processing speed that is one order of magnitude higher (0.5 mL/min versus 50 μL/min typically observed in microfluidic devices [27]). Furthermore, cohesive scaffolds are prepared within a very short time, for the benefit of cell survival, with a one-step procedure, avoiding the need for an additional step that is required to assemble discrete fibers that are produced in similar systems [24,37,38,39].

### 3.2. Blended Microfiber Fabrication

**Figure 3:**
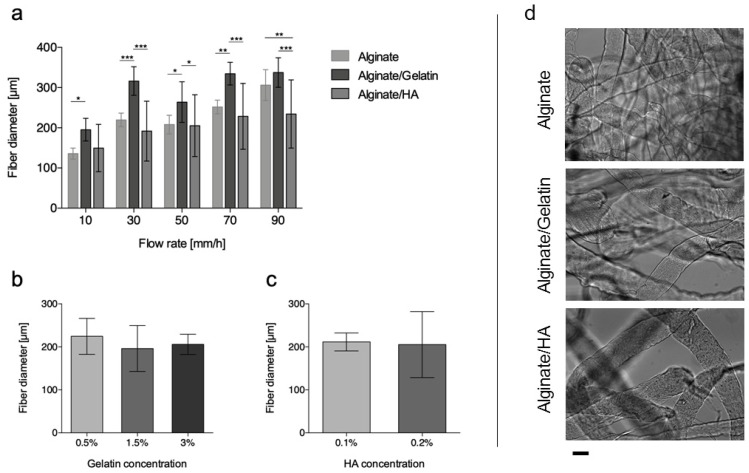
Effects of different parameters on fiber dimensions. (**a**) Effects of solution composition and solution flow rates. Effect of blend components on fiber diameters: for (**b**) gelatin and (**c**) HA blends. (**d**) Optical images of alginate, alginate/gelatin, and alginate/HA microfibers. Scale bar = 200 μm. (* *p* < 0.05, ** *p* < 0.01 and *** *p* < 0.005).

The addition of gelatin or HA, as qualitatively shown in Figure 3d, did not inhibit fiber formation, neither did it affect the general morphology of the microfibers. In the case of gelatin, even when its proportion in the blend was higher than alginate (3% vs. 2%) smooth and homogeneous fibers were obtained.

**Figure 4:**
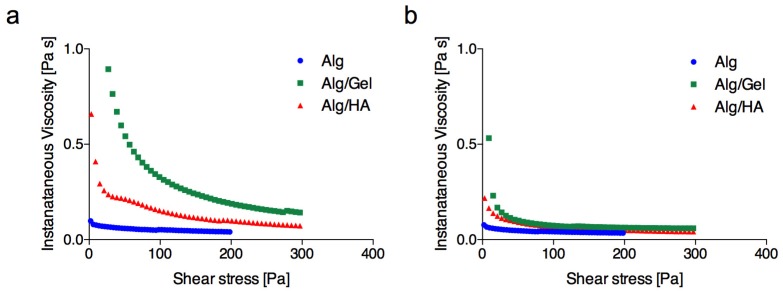
Instantaneous viscosity as a function of shear stress for plain alginate (2% *w*/*v*) and alginate blend solutions (Alg/Gel is composed of 2% (*w*/*v*) alginate and 1.5% (*w*/*v*) gelatin; Alg/HA is composed of 2% (*w*/*v*) alginate and 0.2% (*w*/*v*) HA) at 25 °C (**a**) and 37 °C (**b**).

When increasing solution flow rate, the trend in microfiber diameter for blends was consistent with that observed for pure alginate, but the introduction of gelatin into the polymer solution determined a significant increase of microfiber diameter for all flow rates and concentrations (Figure 3a,b). This finding is consistent with an increase in solution viscosity, as revealed by rheological measurements (Figure 4a), imputable to the increased overall concentration of the solution. 

Contrarily, HA did not have the same effect at both of the concentrations chosen for blending (0.1% and 0.2% (*w*/*v*), Figure 3c) [40,41]. Due to the high molecular weight, HA was added in at lower percentages, and the increase in viscosity at room temperature was less pronounced (Figure 4a).

Rheological measurements (Figure 4) also showed a distinct influence of temperature on the viscosity changes in both blends. While the alginate curve was substantially unchanged, the addition of both gelatin and HA caused a substantial increase in the viscosity at room temperature, while the effect was significantly less noticeable at 37 °C, where the curves of instantaneous viscosity were largely overlapped (Figure 4b).

### 3.3. Microfiber Swelling and Weight Loss 

**Figure 5:**
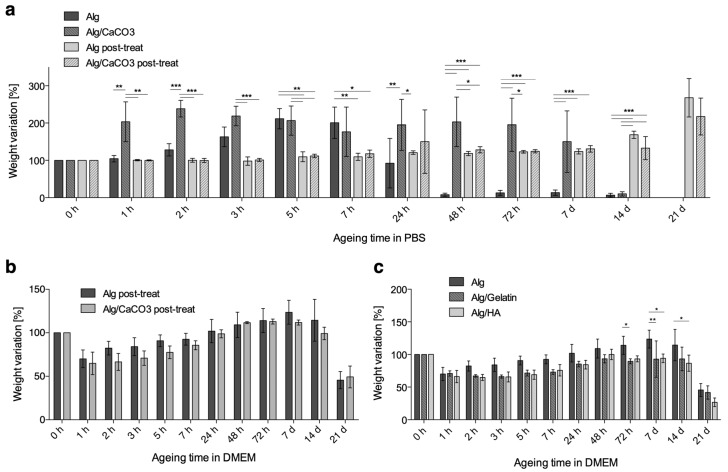
Percentage weight variation after ageing in (**a**) Phosphate Buffer Saline (PBS) and in (**b**) Dulbecco’s Modified Eagle’s Medium (DMEM) for microfibers prepared with 2% alginate solution, 50 mm/h flow rate, and a 24 G needle. (**c**) Percentage weight variation after ageing in DMEM for alginate-blended microfibers prepared with a 2% alginate solution blended with 1.5% gelatin or 0.2% HA (* *p* < 0.05, ** *p* < 0.01, *** *p* < 0.001).

Swelling and weight loss of microfibers in PBS and in the cell culture medium at 37 °C for up to 21 days are shown in Figure 5. Plain alginate patches (Figure 5a,b) without any further crosslinking lost the majority of their weights within the first 48 h.

Based on the rapidity of weight loss and the degree of swelling, it is reasonable to assume that, due to the low concentration of CaCl_2_ a weakly cross-linked shell forms around a nearly liquid core [42]. As the shell breaks because of mechanical stress caused by swelling, the release of the liquid core accounts for the steep weight loss. Accordingly, when CaCO_3_ is added to the alginate solution to create a denser core, a higher persistence of fibers is observed, and the greatest weight loss takes place between 7 and 14 days. When the patches underwent a post treatment by immersion in concentrated CaCl_2_ solution (150 mM), a considerably higher PBS stability was observed, and swelling was still prevailing over degradation after 14 days. This large delay in swelling is likely to be due to both a denser (in shell) and deeper (in core, for the higher chemical gradient) amount of crosslinking. In this case, in fact, no relevant effects of calcium carbonate appeared. 

The effects of post-treatment in a high molarity CaCl_2_ solution also had an important effect on the adhesion between fibers, as patches were more consolidated and resistant to handling. For this latter reason, together with it being less of a threat for cell viability (compared to internal gelation), this method was preferred for its increased fiber stability.

Interestingly, a very different mode of behavior was observed when post-treated patches were aged in complete medium (Figure 5b). Here, alginate patches lost a considerable percent of their weight (around 30%) within the first hour. After this initial contraction, a weight increase was observed for up to 7 days, followed by a progressive loss. As the swelling of ionic gels is controlled by the osmotic pressure in the gel and the elastic reaction of its network [43], it is not surprising to observe different behaviors in volume change after its immersion in solutions with different ionic strengths. In calcium alginate gels, in particular, swelling and degradation are regulated on an exchange mechanism between cross-linked Ca^2+^ ions and Na^+^ ions in the ageing media. When this exchange involves Ca^2+^ that are bound to COO^−^ groups of polymannuronate blocks, the resulting chain relaxation favors swelling, while degradation begins when egg-box-forming Ca^2+^ ions in polyguluronate blocks start to exchange with Na^+^ [44]. 

Accordingly, the different behaviors in swelling and weight loss in PBS and the complete media can be explained by the differences in the types and concentrations of the solutes. As the difference in weight loss stands out when comparing the results in Figure 5a,b, its implications for the cell release profiles should be taken into account.

In Figure 5c, the swelling profile of blended hydrogel is shown. As observed in plain alginate samples, a significant degree of weight loss (around 30%) was measured within the first few hours of incubation, for the three types of microfibers (alginate, alginate/gelatin 1.5% and alginate/HA 0.2%). After this initial contraction, the alginate microfibers started to swell, and they increased in weight for up to 7 days, and they decreased again thereafter, to reveal a progressive dissolution of fibers. Although it occurred to different extents, the sequence of contraction, swelling, and dissolution was observed for all types of microfibers.

### 3.4. Cell Encapsulation in Alginate Microfibers

**Figure 6:**
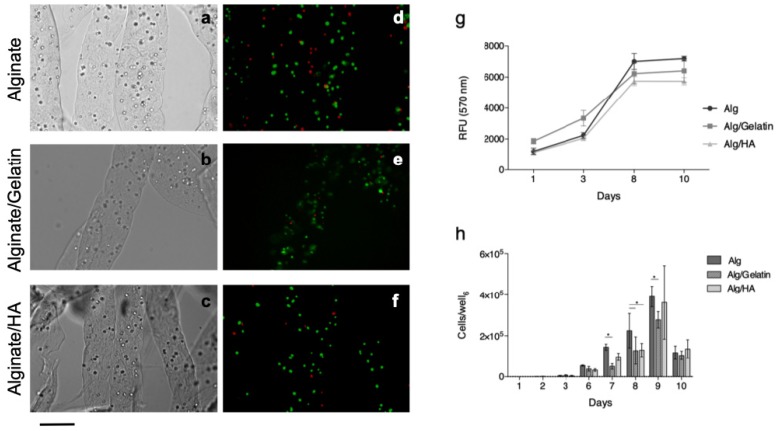
White light (**a**–**c**) and fluorescence (**d**–**f**) micrographs of fibroblast-loaded microfibers. Fluorescence images were taken shortly after embedding and staining, with calcein-AM and propidium iodide. Scale bar = 200 μm. Evaluation of L929 fibroblast behavior after embedding in alginate and blended microfibers: (**g**) Alamar Blue^®^ fluorescence intensities and (**h**) the number of cells released from the microfibers.

Microfibers for cell embedding were produced using a 2% (*w*/*v*) alginate solution with the addition of 1.5% (*w*/*v*) or 0.2% (*w*/*v*) of gelatin and HA, respectively. Under these conditions, embedded cells were distributed as single cells uniformly along the microfibers, as shown in the optical micrographs (Figure 6a–c).

According to staining that was performed shortly after encapsulation (Figure 6d–f), the process allows for a good degree of viability to be preserved in all materials (75 ± 16% for alginate/gelatin blend, 71 ± 6% for alginate/HA blend and 79 ± 6% for plain alginate).

Over longer time spans, alginate-based microfibers were also confirmed to offer an adequate environment for the survival of embedded cells, as demonstrated from Alamar Blue^®^ results (Figure 6g). A progressive increase in fluorescence intensity is observed until day 8, but this is unchanged at the following time point. If this result is compared with the cell release profile (Figure 6h), it can be noticed that a large number of cells are actually released between day 8 and 10. As a consequence, proliferation and Alamar Blue^®^ reduction rely only on a lower number of cells. For all of the formulations, the released cells appeared to be viable, and they were well-adhered to the bottom of the well (Supplementary materials—Figure S2). 

## 4. Conclusions

Designing a hydrogel scaffold for the in situ release of embedded cells raises many challenges, especially in terms of the compatibility of the fabrication with cell survival, adequate mass exchange, and the tuning of degradation profiles. For some types, however, the fiber-encapsulation system that has been designed in this work appears to offer some effective solutions.

Despite its simplicity (it can be built in a short period time, with readily available, inexpensive components), the system that has been designed in this study allows for the rapid (about 3 min) fabrication of large, cell-loaded fibrous scaffolds. The distribution of cells is very regular and homogenous within the fibers and throughout the scaffold. Furthermore, the two-step crosslinking process, by consolidating bonding between the fibers, results in cohesive, easy-to-handle scaffolds. 

In the fibrous patch, only a thin layer of material separates the cells from the environment, and a uniform network of macro-pores is created in the structure as the fibers assemble. This favorable architecture, according to the viability results, appears to be a large benefit in terms of mass-exchange and cell survival, compared to bulk hydrogels.

In addition, the flow-circuit system demonstrated a significant degree of flexibility in terms of material composition, as fiber formation was possible for a relatively large range of parameters. As plain alginate might not always represent the ideal material [45], blending fiber-forming alginates with other molecules or macromolecules appears to be an interesting option for reducing alginate quantities in favor of metabolically degradable components, instructions for cell behavior or tuning for cell release [42]. 

For all of the above-mentioned reasons, the developed fiber-encapsulating system appears to be a very powerful platform for in vivo cell delivery in regenerative medicine, but also, more generally, for in vitro cell culturing. For this latter technique in particular, a simple, widely available and effective technique for preparing effective cell-loaded scaffolds is highly desirable, as it represent an essential tool in the development of 3D cell cultures and more reliable in vitro models for many applications.

## Figures and Tables

**Table 1:** Composition of blends for microfibers patch fabrication.

Sample Label	Final Concentrations (% *w*/*v*)		
	Alginate	Gelatin	Hyaluronic Acid (HA)
Alg	2	-	-
Alg/Gelatin	2	0.5	-
		1.5	
		3	
Alg/HA	2	-	0.1
			0.2

## Supplementary Materials

The following are available online at https://www.mdpi.com/2306-5354/6/2/30/s1, Figure S1: Effect of CaCl_2_ flow rate on microfiber diameter, Figure S2: cells released from microfibers at the bottom of the cell culture wells.
