# Supplementary figures and images for: 3D Encapsulation Made Easy: A Coaxial-Flow Circuit for the Fabrication of Hydrogel Microfibers Patches
Source: Bioengineering (Basel). 2019 Apr 6;6(2):30. doi: 10.3390/bioengineering6020030 (PMC6631674; doi:10.3390/bioengineering6020030)

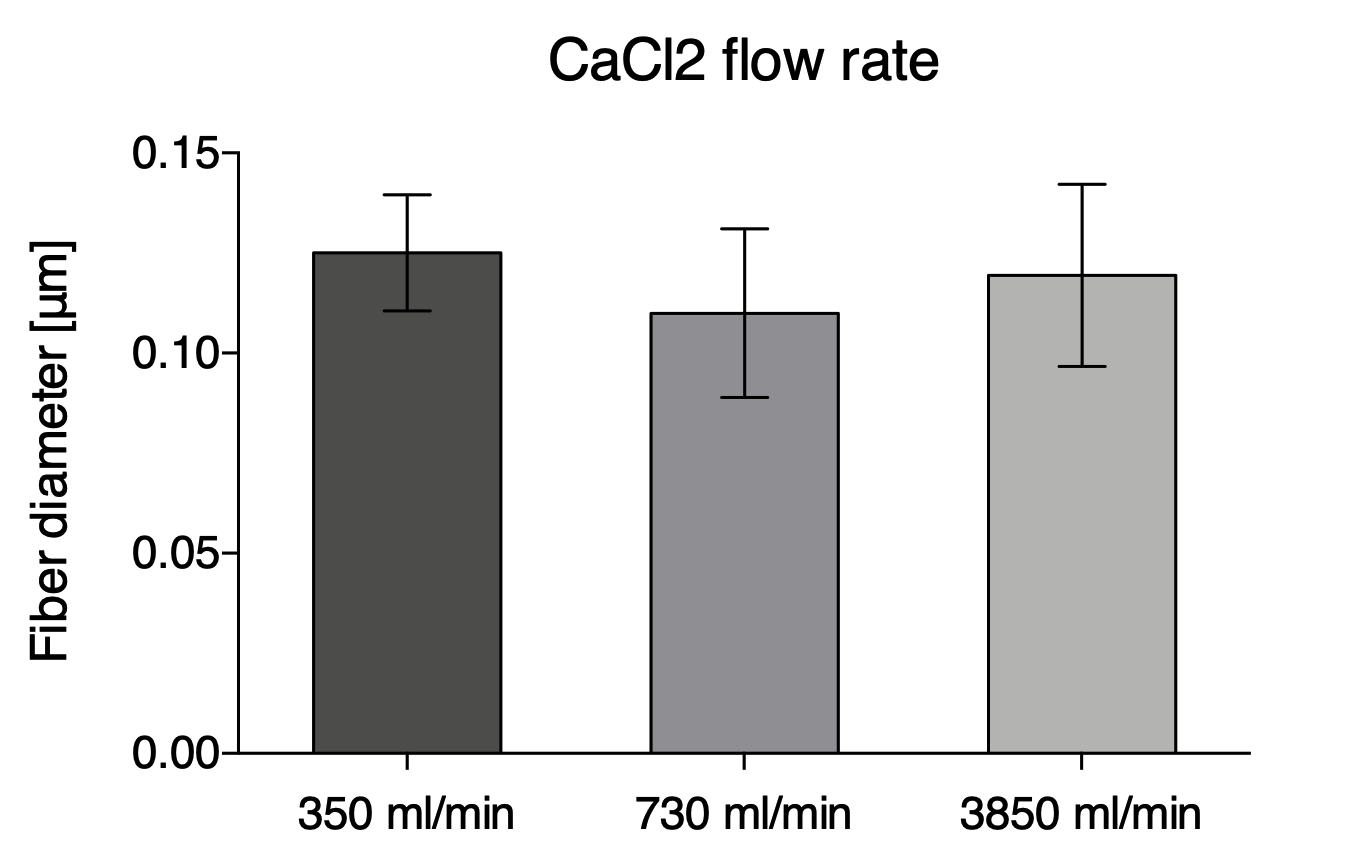

Supplement: Supplementary file 1 [file bioengineering-06-00030-s001.zip › Supplementary 1.tiff]

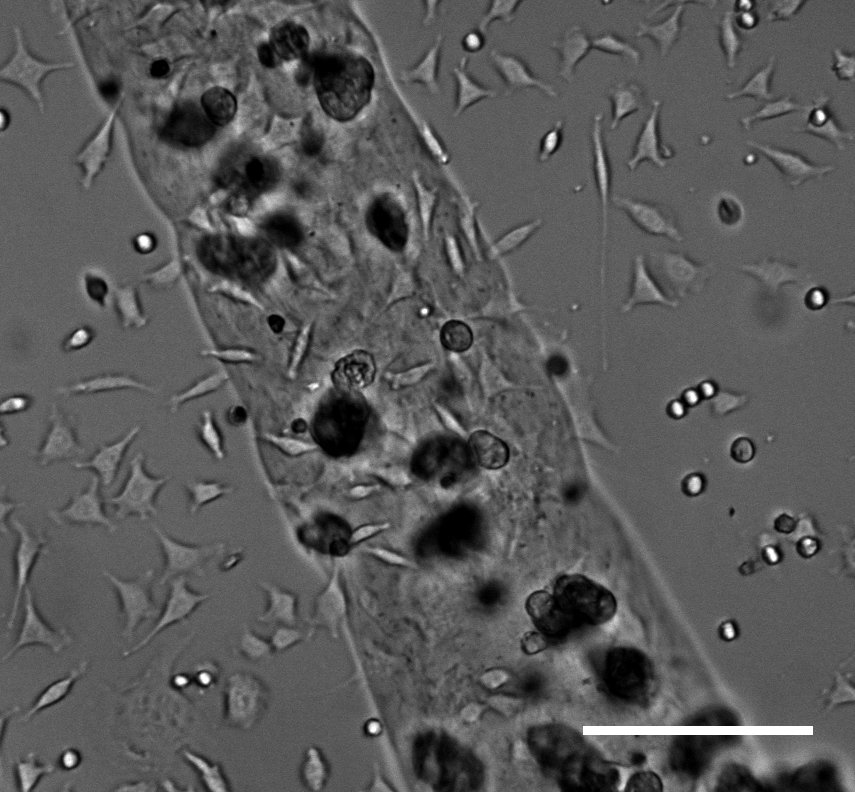

Supplement: Supplementary file 1 [file bioengineering-06-00030-s001.zip › Supplementary 2.tiff]
